# Pancreatic cancer survival analysis defines a signature that predicts outcome

**DOI:** 10.1371/journal.pone.0201751

**Published:** 2018-08-09

**Authors:** Pichai Raman, Ravikanth Maddipati, Kian Huat Lim, Aydin Tozeren

**Affiliations:** 1 School of Biomedical Engineering, Sciences, and Health Systems, Drexel University, Philadelphia, PA, United States of America; 2 Department of Biomedical and Health Informatics, Children’s Hospital of Philadelphia, Philadelphia, PA, United States of America; 3 Center for Data-Driven Discovery in Biomedicine, Children’s Hospital of Philadelphia, Philadelphia, PA, United States of America; 4 Division of Gastroenterology, Department of Medicine, Perelman School of Medicine at the University of Pennsylvania, Philadelphia, PA, United States of America; 5 Stoke Therapeutics, Inc., Bedford, MA, United States of America; Centro Nacional de Investigaciones Oncologicas, SPAIN

## Abstract

Pancreatic ductal adenocarcinoma (PDAC) is the third leading cause of cancer death in the US. Despite multiple large-scale genetic sequencing studies, identification of predictors of patient survival remains challenging. We performed a comprehensive assessment and integrative analysis of large-scale gene expression datasets, across multiple platforms, to enable discovery of a prognostic gene signature for patient survival in pancreatic cancer. PDAC RNA-Sequencing data from The Cancer Genome Atlas was stratified into Survival+ (>2-year survival) and Survival–(<1-year survival) cohorts (n = 47). Comparisons of RNA expression profiles between survival groups and normal pancreatic tissue expression data from the Gene Expression Omnibus generated an initial PDAC specific prognostic differential expression gene list. The candidate prognostic gene list was then trained on the Australian pancreatic cancer dataset from the ICGC database (n = 103), using iterative sampling based algorithms, to derive a gene signature predictive of patient survival. The gene signature was validated in 2 independent patient cohorts and against existing PDAC subtype classifications. We identified 707 candidate prognostic genes exhibiting differential expression in tumor versus normal tissue. A substantial fraction of these genes was also found to be differentially methylated between survival groups. From the candidate gene list, a 5-gene signature (*ADM*, *ASPM*, *DCBLD2*, *E2F7*, and *KRT6A*) was identified. Our signature demonstrated significant power to predict patient survival in two distinct patient cohorts and was independent of AJCC TNM staging. Cross-validation of our gene signature reported a better ROC AUC (≥ 0.8) when compared to existing PDAC survival signatures. Furthermore, validation of our signature through immunohistochemical analysis of patient tumor tissue and existing gene expression subtyping data in PDAC, demonstrated a correlation to the presence of vascular invasion and the aggressive squamous tumor subtype. Assessment of these genes in patient biopsies could help further inform risk-stratification and treatment decisions in pancreatic cancer.

## Introduction

Pancreatic cancer is the third leading cause of cancer related death in the US and is predicted to become the second leading cause of cancer mortality by 2020 [1]. Despite recent advances, the 5-year survival rate remains less than 7% [2]. The majority of patients present with advanced stage disease, and available treatments with FOLFIRINOX or nab-paclitaxel plus gemcitabine chemotherapy provide only modest survival benefit [3,4]. In addition, patients who undergo attempts at curative surgery plus adjuvant chemotherapy still have a very poor 5-year survival rate at roughly 15–20% with 80% of patients relapsing after resection [5]. These poor outcomes highlight the need for novel development of biomarkers to predict patient survival and treatment response with potential linkage to different therapeutic options.

The American Joint Committee on Cancer (AJCC) TNM staging system is currently the most widely used prognostic factor for predicting survival in patients with pancreatic cancer [6]. The system relies primarily on accurate assessment of tumor size, lymph node involvement, and presence of metastasis. In an effort to develop better prognostic tools to stratify patient survival, probability of recurrence, and treatment response, several groups have developed gene expression signatures utilizing microarray datasets derived from pancreatic cancer patients [7–10]. While these signatures performed better than AJCC TNM staging in predicting patient survival, clinical uptake has been lacking, in part because many of these signatures were derived solely from microarray data which does not capture mRNA expression as accurately as RNA-Sequencing and limits the dynamic range for detecting gene expression differences between patient samples [11].

With the advent of next-generation sequencing technologies, it is now possible to obtain a complete picture of the mutational and transcriptional landscape of most tumors. In pancreatic cancer, this has been elucidated through many large-scale studies such as the Cancer Genome Atlas (TCGA) and the International Cancer Genome Consortium (ICGC). These analyses have identified many of the core genetic pathways activated in PDAC and have enabled identification of distinct molecular subtypes associated with differences in therapy response [10,12–16].

Our current study aims to integrate transcriptional analyses from multiple data sets and platforms in order to identify genes with expression profiles predictive of survival in pancreatic cancer patients. To define genes associated with survival we first analyzed RNA-Sequencing and paired survival data from the TCGA database. The resulting genes were then intersected with existing tumor and matched normal datasets to identify genes associated with transformation and with minimal normal tissue expression [17]. The resulting survival associated differentially expressed genes (DEGs) incorporated expression differences from both the tumor and stromal compartments of the tumor–which in combination are thought to more accurately reflect the underlying biology of pancreatic tumors [15,18,19]. This set of genes was then trained on the ICGC pancreatic cancer cohort to identify a 5-gene prognostic signature. Our signature was tested against three other predictive PDAC signatures and performed markedly well in two independent microarray datasets (GSE57495, GSE71729) [8,15]. The gene signature was also found to correlate with vascular invasion on histology and was predictive of survival independent of AJCC stage. Finally, the genes identified were highly associated with molecular features of aggressive pancreatic tumor subtypes.

## Methods and methods

The use of human tissues for immunohistochemistry was approved by the institutional review board at the University of Pennsylvania. Formalin fixed, paraffin-embedded tissues of human PDAC following surgical resection were obtained from the Cooperative Human Tissue Network (CHTN: https://www.chtn.org/) and processed by Molecular Pathology and Imaging Core (MPIC: http://www.med.upenn.edu/molecular/core_morphology.shtml) at the University of Pennsylvania.

### Pancreatic cancer gene list development

TCGA Pancreatic RNA-Sequencing expression data and associated survival data were obtained from the Broad GDAC Firehose site (https://gdac.broadinstitute.org/). RNA-Sequencing data were first filtered to remove genes with general low expression (< 100 counts). When multiple entries were found referencing the same gene, a single representative with the maximum value was selected. These filtering steps dropped the number of candidate genes from 20,330 genes to 12,959 genes. In addition, only samples with both RNA-Sequencing expression and survival data were used for subsequent analysis comprising 178 patients. Samples were split into 2 groups for comparison, those surviving less than 1 year (Survival-) and those surviving greater than 2 years (Survival+). Purity data for PDAC TCGA samples in Survival+/- groups was obtained from Raphael et. al. analysis of the TCGA PAAD dataset [20]. Data was available for 37 of the 47 samples on our survival cohort.

Microarray data for tumor versus normal comparison were obtained from GEO, entry GSE28735 using the *GEOquery* R package [21]. Hugo Gene Symbols were mapped to each probe in the platform (HuGene 1.0 ST) using the probeset annotation as specified in GEO. Analysis was performed on the gene level and for each set of probesets mapping to the same gene the one with the highest maximum value was chosen as the representative gene. This step filtered the number of entries from 28,869 probes to 20,254 genes. The dataset itself were composed of 45 tumor and normal-matched pairs comprising 90 samples in total, with associated survival data. The patients were spread across different grades & stages. The ESTIMATE algorithm was used to determine the relative purity of the 45 tumor samples [22].

For the analysis of the TCGA RNA-Sequencing data the *voom* package in R was initially used to transform the data from counts into values amenable for linear modeling [23]. Following this, the *limma* package in R was used to determine genes that were differentially expressed between Survival- and Survival+ samples. This package was used similarly to determine genes differentially expressed between tumor and normal tissue in the GSE28735 microarray dataset. No initial conversion was needed in this case because data coming from the GEO data repository were RMA normalized and log-transformed allowing for linear modeling. For both comparisons, an adjusted P-value (Benjamini-Hochberg) cutoff of 0.05 and a fold-change cutoff of 1.5 were used to determine differentially expressed genes (DEGs). The two DEG lists were intersected to define the genes expressed in common.

### Methylation analysis

Methylation data for the TCGA PAAD dataset was downloaded from the cBioPortal github (https://github.com/cBioPortal/datahub/tree/master/public). For analyses of the methylation data we first converted the data to M values using the *lumi* package and then used the *limma* package to compare gene methylation profiles between the same Survival+ (28 samples) and Survival- (19 samples) groups used in the differential expression analysis [24]. To call differential methylation we used an adjusted p-value (Benjamini-Hochberg) threshold of 0.05 and additionally had a cutoff of delta beta ≥0.2 or delta beta ≤-0.2.

### Signature development & ROC analysis

To define a survival signature, pancreatic cancer RNA-Sequencing data from the International Cancer Genome Consortium (ICGC) was retrieved. Specifically, we obtained the Australian Pancreatic Cancer data set from the ICGC (https://dcc.icgc.org/releases) encompassing 242 samples profiled on the Illumina HumanHT-12 V4.0 expression beadchip. The ICGC pancreatic cancer data were filtered to the set of genes (707 genes) stemming from the intersection of the TCGA Survival Analysis and tumor versus normal comparison (GSE28735). From here Survival+ (42 samples) and Survival- (61 samples) groups were defined in the ICGC data according to the same guidelines employed previously with the TCGA survival analysis, confining the analysis to 103 samples. S5 Fig shows a summary of the clinical data between the two groups. We then used a sampling based method to iteratively (10 iterations) pull 15 samples (with replacement) from each group (Survival+ and Survival-) and used the *limma* package to determine DEGs (P-value < 0.05). Genes that were repetitively found significant in more than 5 iterations (>50%) were included in the signature. After this step, there were 8 genes in the signature. From here, the signature was further filtered to remove genes that were highly correlated to one-another. This was done manually through visual inspection of the correlations, taking into account the predictive power of each gene, and resulted in the removal of just 3 genes. Subsequently, the final signature (5 genes) was tested via receiver operating characteristic (ROC) analysis on the ICGC data (42 Survival+ and 61 Survival- samples). The validity of signature was further established using ROC analysis on 2 separate pancreatic microarray datasets in the GEO data repository, GSE57495 (63 samples, 12 Survival-/17 Survival+) and GSE71729 (357 samples, 41 Survival-/15 Survival+). Both datasets were downloaded using the *GEOquery* package in R. To perform ROC analysis each dataset was split into Survival+ (alive greater than 2 years) and Survival- groups (survival of less than 1 year), similar to thresholds employed in the discovery dataset and the ICGC data set. ROC analysis was also performed on all three data sets using publicly available “gene signatures” for comparison [7–9,25]. Area under the curve (AUC) calculation was ascertained using the *AUC* package in R. For all three datasets 5000 random signatures composed of 5 genes were also generated and signature scores were calculated. This was then used to generate a distribution of AUC’s, the null distribution, to compare with our signature AUC with. In addition to ROC analysis, Kaplan-Meier survival analysis was performed for both validation studies as well as the ICGC data using the signature to delineate groups. In order to create groups for the Kaplan-Meier analysis each dataset was iteratively split into two groups after sorting samples by the ordered signature score. For each of these splits corresponding P-value was calculated by the Mantel–Haenszel test and the lowest P-value was then chosen as the optimal breakpoint. As this method suffers from an increased rate of false-positives a Benjamini–Hochberg correction is applied to reflect the presence of multiple hypotheses testing. To prevent potential batch effects, samples from all three datasets were normalized to a set of housekeeping genes (*TBB*, *ACTB*, *UBC*, *PPIA*, and *GUSB*).

### Correlation to subtype classification

To classify samples according to the Bailey molecular subtypes from studies GSE71729 and GSE57495 we used the defined gene signatures for each subtype and calculated the sum of the standardized gene expression measures for each of the four molecular subtypes and then normalized it by the number of genes in the signature [12]. The class with the maximum normalized standardized score was chosen as the assignment for that sample.

### Multivariate testing of signature score with clinical parameters

In order to test whether the signature was able to accurately determine survival independent of other clinical variables a Cox proportional hazard model was used taking into account tumor grade, sex, and age. This was performed on the ICGC dataset, comprising 237 samples (after removing missing data).

### Visualizations and statistical analysis

Volcano plots, scatter plots, boxplots, and ROC curves were generated using the *ggplot2* package. Venn diagrams were generated using the *VennDiagram* package in R (Version 3.3). Subsequently, certain images were then amended and updated in Adobe Illustrator (AI) [26,27]. All statistical analysis and data processing were performed in the R statistical language. Full code and detailed descriptions of all packages and data sources required for this analysis can be found in a Github repository (https://github.com/PichaiRaman/PDACSurvivalAnalysis).

### Immunohistochemistry

Formalin fixed, paraffin-embedded tissues of human PDAC following surgical resection were obtained from the Cooperative Human Tissue Network (CHTN: https://www.chtn.org/) and processed by Molecular Pathology and Imaging Core at the University of Pennsylvania. Only samples with accompanying pathology reports from a trained pathologist were used for analysis (n = 10). Tumor stage, differentiation status, and histological evidence of vascular invasion were extracted from the pathology reports (S5 Table). Tissues sections were deparaffinized, hydrated, and immersed in 1x R-buffer (Electron Microscopy Sciences) for epitope retrieval in a pressure cooker. Endogenous peroxidase activity was quenched in 3% hydrogen peroxidase for 15 minutes, and slides were then incubated in 0.3% triton-x100 in PBS with 5% normal donkey serum to block nonspecific immunoreactivity. The anti-ADM antibody (1:20, R&D Biosystems, AF6108), anti-ASPM antibody (1:500; Novus Biologicals, NB100-2278), anti-DCBLD2 antibody (1:50, Sigma-Aldrich, HPA016909), anti-E2F7 antibody (1:500, Abcam, ab56022), or anti-KRT6a antibody (1:100, Sigma-Aldrich, SAB2700299) was applied and incubated at 4c overnight followed by staining with appropriate biotinylated secondary antibodies (1:200, Jackson ImmunoResearch). Slides were developed with the DAB peroxidase substrate kit (Vector laboratories, SK-4100) and counterstained with hematoxylin. Patterns of staining were evaluated and quantified using the histological score (H-score) [28].

## Results

### Generation of a tumor gene expression profile that correlates with survival in pancreatic cancer

To determine which genes expressed in pancreatic cancers are associated with survival, the TCGA RNA-Sequencing pancreatic cancer data set was segregated into two groups, one with poor survival (Survival-), and another with better survival (Survival+). With regard to survival time, Survival- groups consisted of patients living less than one year (28 patients) whereas Survival+ groups corresponded to patients living greater than 2 years (19 patients). The split (a full quartile difference) was made at the following intervals to maximize the survival differences in time (1 year) while also ensuring a reasonable number of samples in each group, facilitating detection of differentially expressed genes (DEGs) with potentially low effect sizes (S1 Fig). Aggregate statistics around clinical data between both groups revealed no significant differences in age based on the Kolmogorov-Smirnov test (p-value = 0.39) or gender using the fisher test (p-value = 0.34). In contrast, based on the fisher test, stage and tumor grade were significantly different (p-value = 0.01 and 6x10^-4^, respectively) (S2 Fig). Most Survival- patients were grade 2 whereas many Survival+ patients were more likely to be grade 1. This was not surprising given that tumor grade has been shown to be a marker of survival [29].

**Fig 1:**
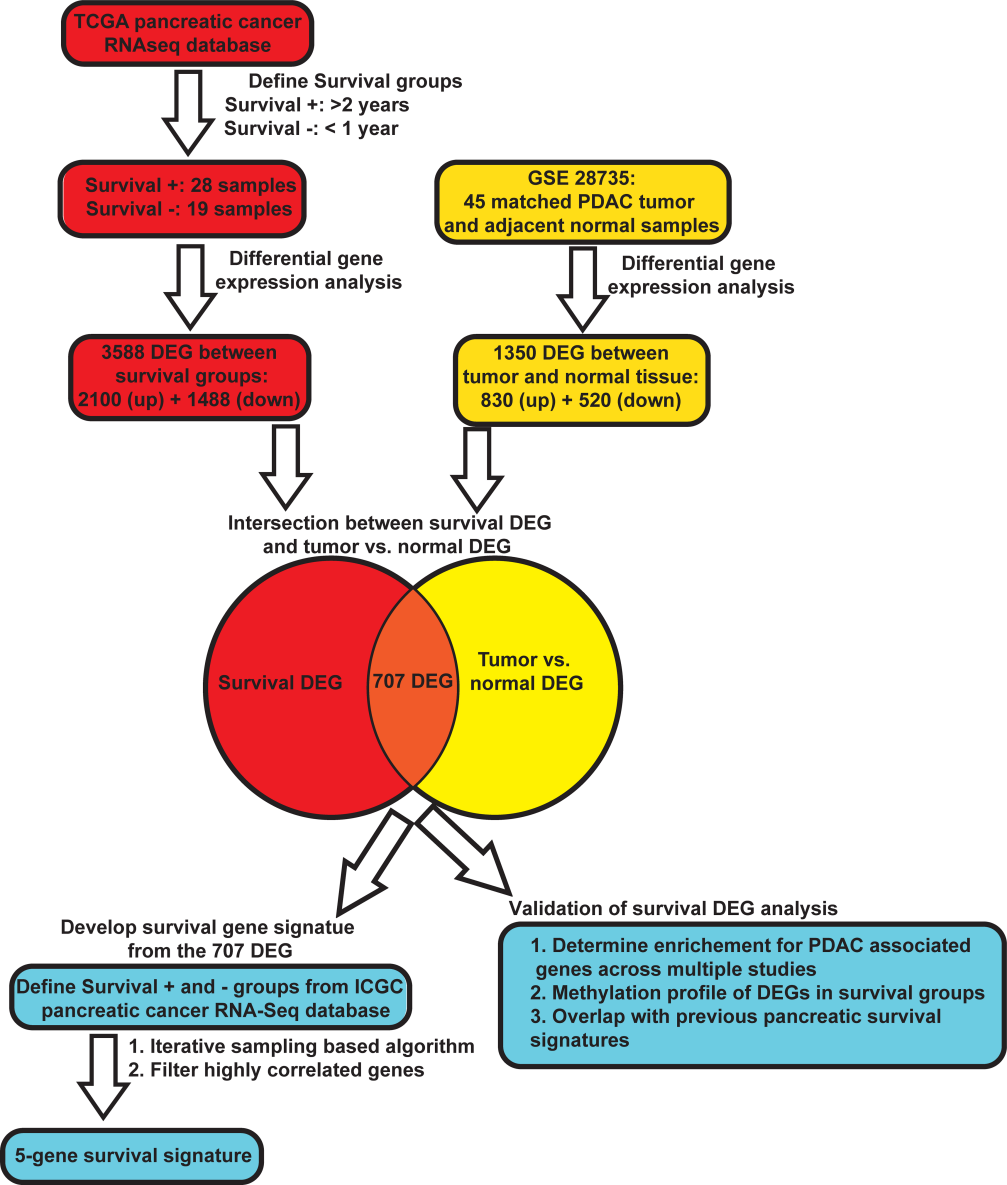
Survival based gene expression gene analysis in PDAC. Flow diagram depicting analysis pipeline to identify 707 differentially expressed genes (DEG) between Survival- and Survival+ groups with subsequent analysis to determine a survival signature.

**Fig 2:**
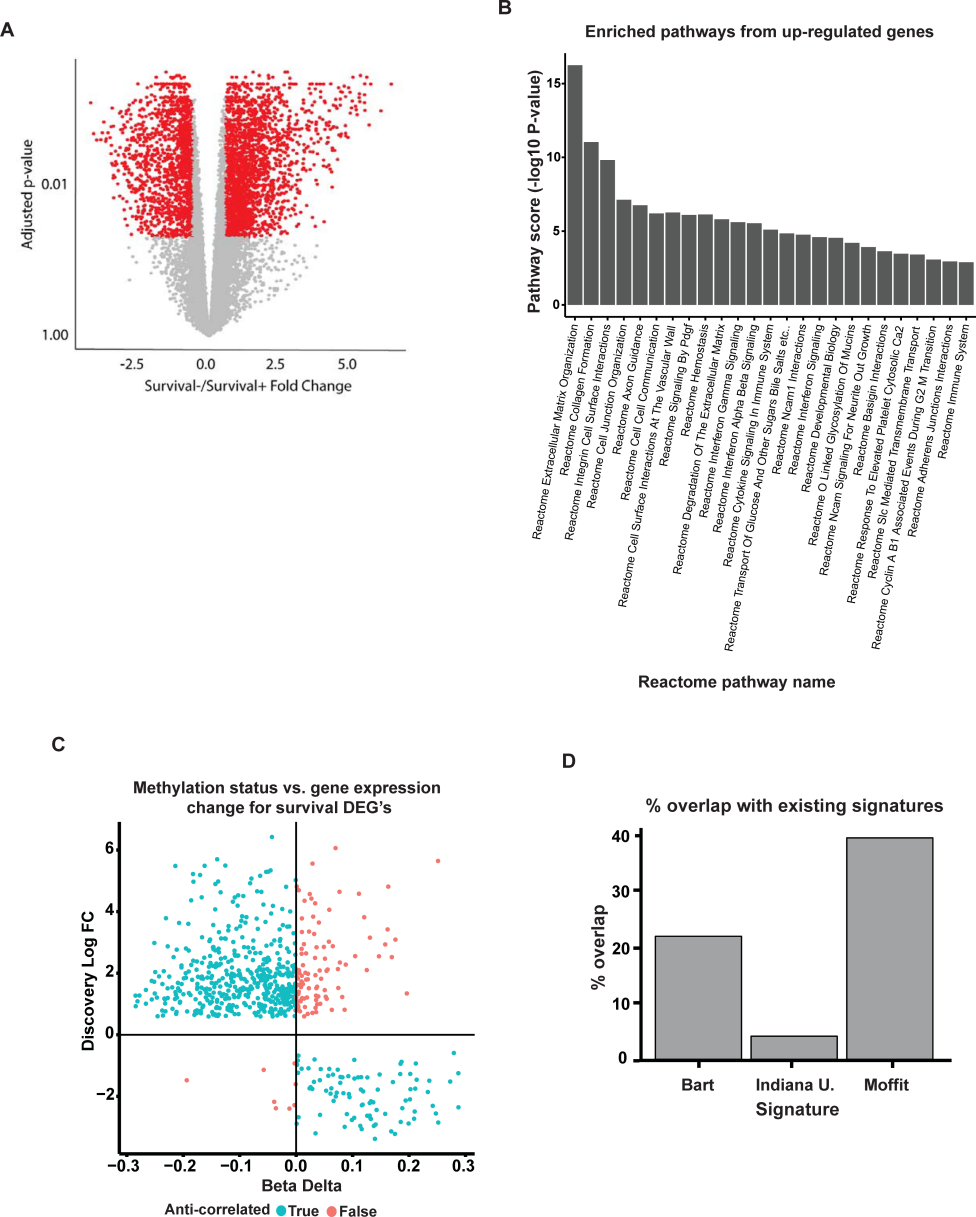
Validation of survival DEG list. (A) Volcano plot highlighting genes associated with survival from TCGA dataset. (B) Bar graph depicting the top 26 enriched pathways based on Reactome pathways analysis of the 602 up-regulated gene from the 707 DEG list. (C) Scatter plot of log fold change of differentially expressed genes vs delta beta of differentially methylated genes between Survival- and Survival+ samples. False indicates methylation status opposite of predicted for gene expression change (total of 31 genes). True indicates concordance between methylation status and gene expression change (total of 676 genes). (D) Bar chart showing percentage overlap of Pancreatic Cancer DEG list with indicated published signatures.

TCGA samples are derived from bulk tumor tissue which contains tumor cells, stroma, and normal tissue contamination. While this could confound tumor-cell specific analyses, recent evidence suggests that therapy response and aggressiveness in many tumors, including pancreatic tumors, derive from the combination of tumor and stromal cell composition in the tumor [15,18–20,30]. Thus, to incorporate gene expression differences in both the tumor and stromal cells we compared the RNA expression profiles of the bulk tumors between the survival groups. We identified a total of 3,588 DEGs (Fig 1). In total 2,100 genes were up-regulated and 1,488 were down-regulated (fold-change of 1.5 and an adjusted p-value of 0.05) in the Survival- cohort compared to Survival+ (Fig 2A).

Although the composition of the tumor stroma is a contributor to survival we wanted to ensure our analysis of Survival+ and Survival- did not directly correlate with the amount of stroma (i.e. tumor purity). To examine this relationship, we obtained data from Raphael et. al. in which they classified PDAC TCGA samples into high and low purity based on several criteria [20].We found that, of the 37 samples in our survival cohort that were classified, more than twice as many Survival- tumors had high purity (19 high purity compared to 8 low purity), whereas Survival+ tumors had an equal number of low and high purity tumors (S3A Fig). While the sample size was small, there was not a high degree of association between stromal content and poor survival (Survival- group) in this cohort. Additionally, we performed the same survival analysis only using the high purity Survival- and Survival+ group and compared the log fold change of the 3,588 DEG. There was a correlation of 0.68 corresponding to a p-value of < 2.2 x 10^−16^ for this set of genes between the two analyses (S3B Fig). Although some degree of correlation was expected, given this subset of high purity samples represents half of the original samples used, the high degree of correlation and the similar direction of fold-changes suggest that using low purity samples did not greatly impact the result except for increasing statistical power.

To identify genes associated with transformation we performed a tumor versus normal comparison on a separate dataset (GSE28735) from the Gene Expression Omnibus (GEO) resource. This study, which encompasses many histologies, has much higher tumor purity, with a median of 0.63 (95% CI: 0.60–0.67), based on the ESTIMATE algorithm (S4A Fig) than the TCGA, which is 0.35 (95% CI: 0.32–0.38). Hence, intersecting this data has the added benefit of potentially removing some of the stromal contamination. For this data analysis, we compared the 45 tumors to their matched normal tissue. Using the same fold-change and adjusted p-value cutoffs, we derived 1350 DEGs consisting of 830 up-regulated and 520 down-regulated genes (S4B Fig), which likely represent gene changes associated with malignant transformation. We then performed an intersection of the two lists to identify which genes were associated with malignant transformation, survival, and aberrantly expressed in reference to normal tissue. After removing genes that moved in opposite directions in the two comparisons (S4C Fig), we identified 707 potential genes of interest (Fig 1 and S1 Table). Specifically, 602 genes were found to be up-regulated in tumor and associated with poor survival whereas 105 genes were down-regulated in tumor and associated with improved survival with only 32 or 4.3% of the list not following the trend (S1 Table).

### Validation of survival based DEG list

To assess the ability of our approach in detecting cancer-related gene expression changes that reflect the underlying biology of PDAC, we first compared our list to those found in the public gene database, mSigDB, and gene signatures found in the literature. Our list identified several genes that have been found to be activated in pancreatic cancer such as MET, MAP4K4, and ITGA2 [31,32]. To determine if the enrichment for PDAC associated genes in our list was statistically significant, we compared it to a gene set from a meta-analysis performed to determine high-confidence pancreatic cancer associated genes across multiple studies [33]. Of the 357 genes found up-regulated in PDAC, 92 were in our list of 602 genes associated with poor survival corresponding to a p-value of 6.2 x 10^−68^ based on the hypergeomtric test. Similarly, of the 202 genes found down-regulated in PDAC, 12 were in our list of 105 genes associated with better survival corresponding to a p-value of 5.6 x 10^−11^.

Next, we performed pathway analysis on our gene list and identified 46 genes sets that are highly up-regulated in pancreatic cancer based on the Reactome database, including *Extracellular Matrix Organization* (adjusted p-value of 6.91x10^-15^) and *Integrin cell surface interactions* gene sets (adjusted p-value of 5.63x10^-^10) (Fig 2B and S2 Table). These pathways are known to regulate stroma formation in PDAC, which in turn influences the aggressiveness of the phenotype [34]. In addition, axon guidance, platelet-derived growth factor, and interferon-gamma signaling pathways were also found to be highly up-regulated in concordance with the literature [35–37].

We next sought to determine if differences in methylation of our genes could explain the differential gene expression between survival groups. Using the TCGA methylation profiles of the same Survival- (28 patients) and Survival+ (19 patients) cohorts, we found that of our 707 genes, 676 were also found to be differentially methylated. Assuming that gene expression is correlated inversely with methylation status, we found that in 83% of detected genes, methylation patterns were highly consistent with fold changes in expression (Fig 2C and see S3 Table).

To determine the extent of overlap between our DEG list and previous pancreatic survival signatures we compared the genes from our discovery analysis with a prognostic 15-gene signature from the Moffitt Cancer Center, a 36-gene prognostic signature from Barts Cancer Institute, and a 48-gene pancreatic cancer angiogenic signature developed at the Indiana University School of Medicine [8,9,25] (Fig 2D). Analysis of the overlap using the hypergeometric test revealed that our list captured many genes from the Moffit (p-value of 2.02x10^-6^) and Barts (p-value of 5.90x10^-6^) signature, and several genes from the angiogenesis gene list from Indiana. Additionally, many genes not captured in any of the previous signatures were also identified; suggesting the potential for novel PDAC associated genes to interrogate.

### Generation of a gene signature predictive of patient survival

From our list of 707 survival associated DEGs, we next sought to identify a set of genes from our list that could accurately predict differences in patient survival (Fig 1). This was accomplished using the ICGC PDAC dataset to establish a training set of Survival+ and–samples (S5 Fig). Employing a sampling-based approach, we derived a 5-gene expression panel significantly associated with survival (Table 1 and S6 Fig). Multivariate testing of the 5-gene signature in the ICGC PDAC dataset found that the signature is predictive of survival (p = 1.3x10^-10^) independent of age, grade, and sex (S4 Table).

**Table 1:** 5-Gene pancreatic cancer survival signature.

Gene	P-value	Direction	Chromosomal location	Gene Description
ADM	2.29x10^-4^	Up	11p15.4	Adrenomedullin
ASPM	2.35x10^-6^	Up	1q31	Asp (abnormal spindle) homolog, microcephaly associated
DCBLD2	6.95x10^-6^	Up	3q12.1|3	Discoidin, CUB and LCCL domain containing 2
E2F7	7.37x10^-5^	Up	12q21.2	E2F transcription factor 7
KRT6A	2.51x10^-4^	Up	12q13.13	Keratin 6A

### Gene signature correlates with vascular invasion and aggressive tumor subtypes

**Fig 3:**
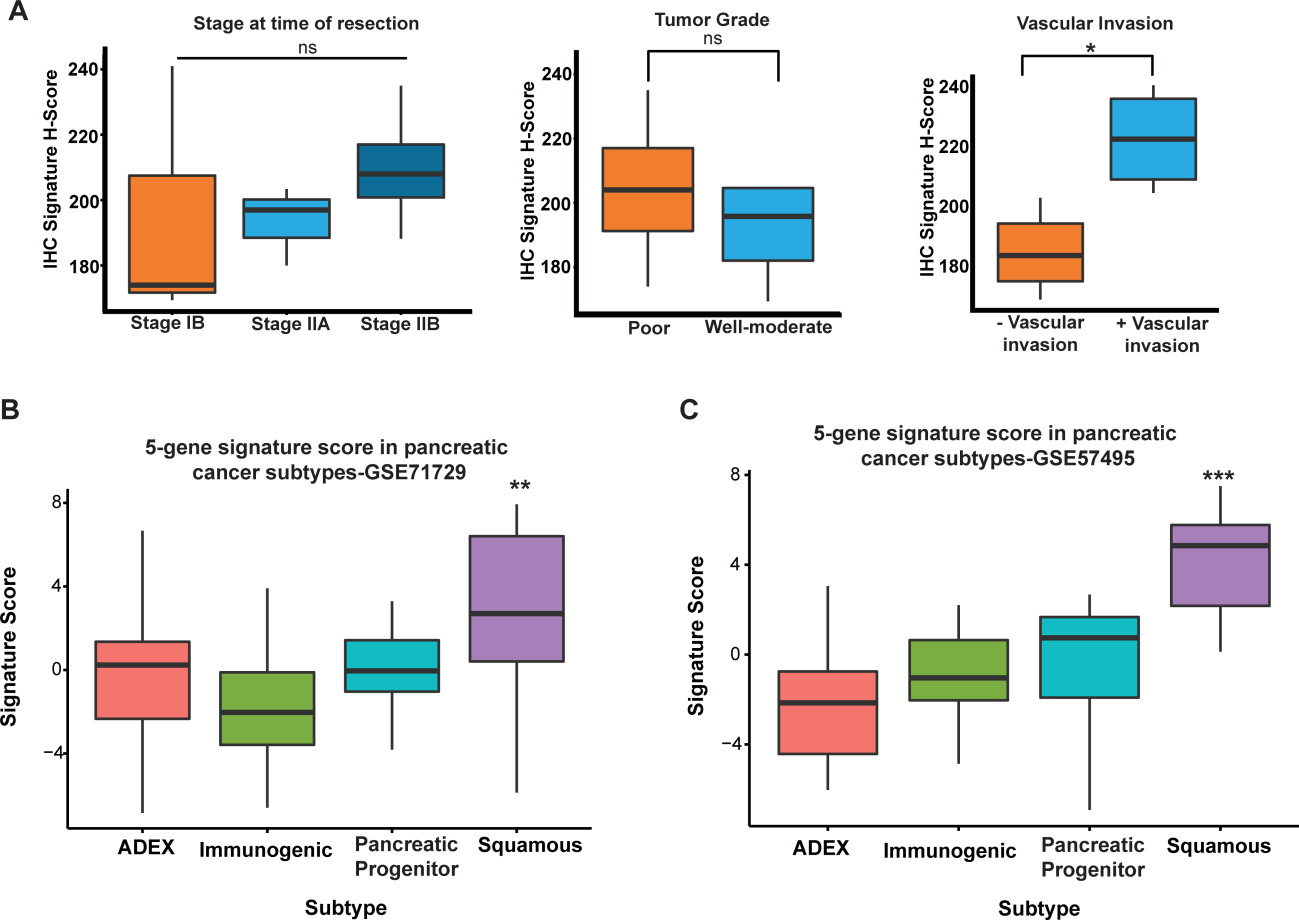
5-gene signature captures histological and molecular features of aggressive PDAC. (A) Box plots showing composite H-score from immunohistochemistry staining of human PDAC samples for ADM, KRT6a, ASPM, DCBLD2, and E2F7 with samples grouped based on AJCC stage, differentiation status, and presence of vascular invasion on histology. N = 10 human PDAC tumor samples. (B) Signature score boxplot versus GSE71729 and (C) GSE57495. *p = 0.01381 and ns = non-significant based on t-test. **p = 4.6 x 10^−8^ and ***p = 7.1 x 10^−7^ based on Anova analysis.

To determine the relationship of the 5-gene signature to histological features of PDAC we examined expression of the 5 genes using immunohistochemistry (S7 Fig) in a panel (n = 10) human pancreatic tumors obtained following surgical resection for either AJCC stage I (3/10) or II (7/10) tumors. Samples were comprised of well-to-moderate (6/10) and poorly-differentiated tumors (4/10) with histological evidence of vascular invasion in 4/10 tumors. Longitudinal survival data was not available for this dataset. A composite H-score (which normalizes for tumor cellularity differences between samples) was calculated for IHC staining (S7 Fig) for each of the 5 genes in our signature [28]. Comparison of scoring between tumor samples found no correlation between our signature and tumor grade or stage (Fig 3A). Interestingly, the tumors with the highest signature score were those where tumor histology revealed evidence of vascular infiltration (Fig 3A) suggesting that our signature may correlate with invasive phenotypes that are seen on histology but not reflected in the AJCC stage [38].

We next sought to determine if our signature could capture the survival differences predicted by recent pancreatic subtype classification systems [12]. Utilizing two independent gene expression datasets (GSE71729 and GSE57495), we compared the signature score to subtype classification and found the median signature score was significantly higher in the squamous tumors (Fig 3B and 3C) [12].

### Gene signature provides improved survival prediction

**Fig 4:**
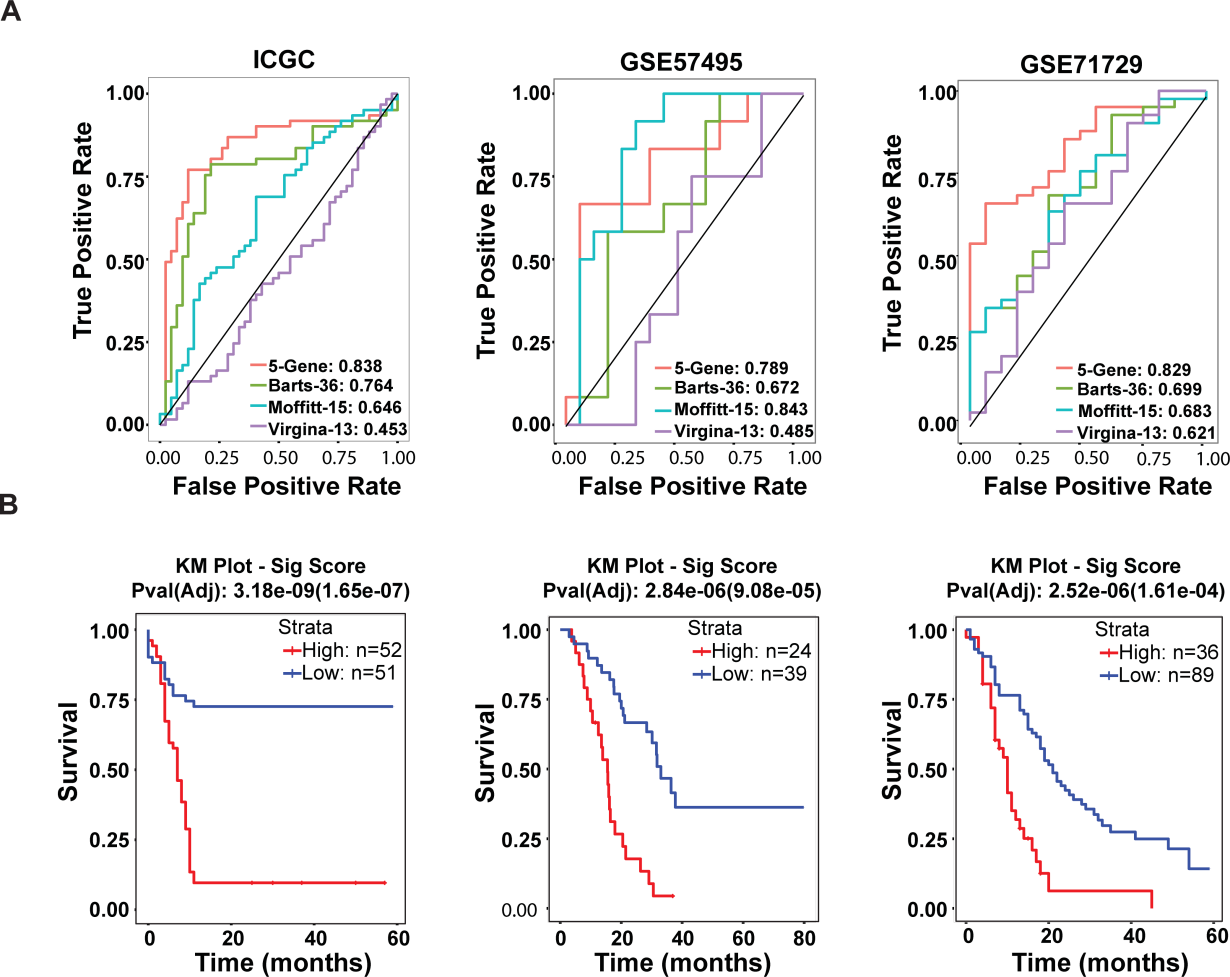
5-gene signature enhances prediction of patient survival in PDAC. (A) ROC curve demonstrating predictive power of pancreatic survival signature in the Pancreatic ICGC (left), GSE57495 (middle), and GSE71729 (right) datasets. (B) Kaplan-Meier plot demonstrating predictive power of pancreatic survival signature in Pancreatic ICGC (left), GSE57495 (middle), and GSE71729 (right) datasets.

We next compared our 5-gene signature to previous pancreatic cancer survival signatures using ROC analysis [7–9,25]. Using these signatures to classify patient survival in the ICGC pancreatic cancer dataset (Fig 4A), we found that our 5-gene signature had a significantly better AUC. This was not surprising considering that our signature was derived using the ICGC cohort, so we also performed ROC analysis on 2 separate pancreatic microarray datasets in the GEO data repository, GSE57495 (63 samples, 12 Survival-/17 Survival+) and GSE71729 (357 samples, 41 Survival-/15 Survival+), which have been used previously to predict survival in PDAC [8,15]. In both datasets, our signature had better ROC characteristics with an AUC of .79 and .83 respectively (Fig 4B). The exception to this was the 15-gene Moffitt Cancer Center signature, which had a better AUC than our signature in GSE57495. However, this was the data set used to derive their initial signature. To account for multiple testing in comparing gene signatures, we also generated 5000 random 5-gene signatures from each data set then compared to our own. We found that in all 3 datasets, our signature significantly (P < 0.005) outperformed randomly generated signatures (S8 Fig). In addition to AUC, we also performed Kaplan-Meier analysis using our 5-gene signature in the ICGC, GSE57495, and GSE71729 datasets and found that our signature could predict survival differences of ≥ 12 months in all three data sets (Fig 4B).

## Discussion

In the current study, we performed an integrative analysis of pancreatic gene expression data derived from the TCGA, ICGC, and Gene Expression Omnibus (GEO) to derive a 5-gene expression signature that predicts overall patient survival. The signature stratifies patients into short (less than one year) and long (greater than two years) survivors. Importantly, the association with survival is independent of AJCC TNM staging, age, gender, and other commonly used clinical factors. Immunohistochemical analysis of human pancreatic tumor samples suggests that our signature is correlated with vascular invasion which is indicative of more aggressive tumor phenotypes [38]. Additionally, our signature was associated with the squamous subtype of PDAC, which is known to have a poor prognosis. Finally, our signature outperformed previously reported signatures across the datasets we tested. From a personalized medicine standpoint, our signature offers a small set of genes that could be readily tested in patient biopsy samples to help risk-stratify patients and inform treatment decisions. However, this will require further validation in larger prospective studies.

While various gene signatures have been developed to predict patient survival in PDAC, these studies were single center and often utilized microarray datasets, which limits the ability to capture the heterogeneity in gene expression that exists in pancreatic tumors. Our integrative approach capitalizes on the diverse patient populations present in the TCGA and ICGC datasets and extends the dynamic range of detectable gene expression changes through analysis of RNA-Sequencing data. Additionally, our initial 707 DEG list identified many of the genes present in these separate studies. Thus, our analysis could capture the heterogeneity in gene expression changes associated with patient survival in PDAC. Importantly, examination of methylation patterns in short and long survivor groups captured a large fraction of the genes in our DEG list and were consistent with the detected fold changes in expression. This suggests that survival differences among patient groups may in part be regulated at the epigenetic level.

In addition to functioning as predictors of patient survival, the genes in our signature also have important roles in the underlying biology of PDAC and other cancers. This may explain their association with poor patient survival, vascular invasion, and correlation with the aggressive squamous PDAC subtype. *ADM* is a multi-regulatory peptide known to regulate pancreas function through direct effects on insulin secretion in ß-cells and amylase secretion in acinar cells. In the setting of pancreatic cancer, increased circulating levels of this hormone are associated with poor prognosis. In part, this effect is mediated through secretion into exosomes that then directly act on adipocytes to increase lipolysis leading to cachexia and on ß-cells resulting in diabetes [39–41]. It is also thought to regulate angiogenesis in PDAC and is secreted in response to hypoxia leading to increased invasiveness [42–44]. *ASPM* is a centrosomal protein that normally regulates neural development and brain size [45]. In PDAC, gliomas, ovarian cancer, and hepatocellular cancer it is up-regulated and associated with poor survival [46–49]. In the context of PDAC, *ASPM* promotes Wnt activity to regulate cancer stemness and thus enhances tumor progression [49]. The roles of *DCBLD2*, *E2F7*, and *KRT6A* have not been explored in PDAC but these proteins are known to have context dependent effects in various cancers. *DCBLD2* is a neruopilin-like membrane protein that modulates *PDGFR-B* and increases during vascular injury. In gastric cancer its down-regulation leads to progression however in glioblastoma, colorectal cancer, and lung cancer it is up-regulated and associated with increased tumorigenesis and invasion [50–53]. *E2F7* is a known cell cycle regulator that is associated with poor survival in squamous cancers, upregulates c-MYC in various cancer cell lines, and induces tamoxifen resistance in breast cancer [54–56]. Finally, *KRT6A* is a cytoskeletal scaffolding protein whose increased expression is associated with improved survival in breast cancer but portends a worse prognosis in lung cancer and is associated with squamous differentiation [57–59].

While our study provides an improved survival gene signature in PDAC, the analysis was primarily derived from patients with surgically resectable disease and annotated survival data which may limit the prognostic value of our signature to a subset of the PDAC patient population. Future studies aimed at replicating our findings in larger PDAC patient cohorts will be needed. In addition to limitations in patient selection, our studies were also confined to the analysis of RNAseq and microarray data derived from bulk primary tumor. In the TCGA PAAD dataset, samples have high stromal content thus limiting direct assessment of tumor cell specific gene expression. However, our approach sought to incorporate gene expression differences reflective of both the tumor and stromal compartments. Recent evidence suggests that in addition to the tumor cells, the composition of the stroma (rather than the actual amount of stroma/tumor cell purity) is critical to the underlying biology of the tumor [15,18–20]. This is supported by our finding that tumor cell purity is not predictive of survival and did not significantly influence our survival associated DEGs. Thus, our approach sought to capture the survival associated gene expression of the bulk primary tumor. In addition, a list that includes tumor and stromal gene expression is likely clinically relevant as genomic analysis is often performed on bulk tumor samples.

## Conclusions

In the current study, we perform an integrative analysis of large-scale pancreatic gene expression datasets to define a gene signature predictive of survival. Our analysis identified a 5-gene panel that performed well against previous signatures across multiple datasets and captures subtype-specific differences in patient prognosis. Further testing in a larger cohort will be needed to validate the prognostic value of our gene signature. We hope that our *in silico* approach enables accurate prediction of patient survival from biopsy specimens in PDAC and provides a framework for similar assessments in other cancers.

## Supporting information

S1 Fig Distribution of survival times and creation of groups in TCGA (discovery) dataset. (PDF)

S2 Fig Population cohort characteristics of survival groups form TCGA dataset. Clinical summary information of age, stage, grade, and gender differences between the Survival- and Survival+ groups. Stage and tumor grade were significantly different (p-value = 0.01 and 6x10-4, respectively).
(PDF)

S3 Fig Impact of tumor cell purity in TCGA PAAD dataset on survival based gene expression analysis. (A) Bar graph showing breakdown of the available high and low tumor cell purity samples present within the Survival + and—groups. (B) Correlation of Survival associated DEGs in high purity PAAD samples to all PAAD samples in the survival cohort. Correlation between DEGs was 0.68 with p < 2.2 x 10^−16^.
(PDF)

S4 Fig Tumor versus normal pancreatic tissue comparisons to identity genes likely relevant to malignant transformation. (A) Analysis of tumor cell purity in GSE28735 using the ESTIMATE algorithm. (B) Volcano plot of tumor versus normal pancreatic tissue from the GSE28735 dataset. (C) Scatter plot of log fold change from tumor versus normal comparison and log fold change from survival analysis with signature genes selected.
(PDF)

S5 Fig Population cohort characteristics of ICGC training set. Clinical summary information of (A) grade, (B) gender, (C) age, and (D) stage for the 103 samples in the Australian pancreatic cancer dataset from the ICGC database.
(PDF)

S6 Fig Generation of a 5-gene survival signature. (A) Number of genes significant in N iterations of differential expression analysis. (B) Correlation matrix of genes highly predictive of survival to be considered for signature.
(PDF)

S7 Fig Representative IHC images of human Pancreatic tissue samples stained for ADM, KRT6a, ASPM, DCBLD2, and E2F7. Left column represents tumor tissue from sample ID 716048 which had evidence of vascular invasion. Right column represents tumor tissue from sample ID 1021055 which did not have evidence of vascular invasion. Scale bar is 50μm for all images.
(PDF)

S8 Fig Comparison of 5-gene survival signature to random gene signatures. Comparison of null distribution of AUC values to AUC of pancreatic survival signature based on Pancreatic ICGC (left), GSE57495 (middle), GSE71729 (right) datasets.
(PDF)

S1 Table Differentially expressed gene list from survival and tumor vs. normal comparisons. DEG list of 707 genes derived from intersection of genes differentially expressed between pancreatic Survival+ and Survival- patients and from pancreatic tumor versus normal comparison.
(XLS)

S2 Table Pathway analysis of pancreatic cancer DEG list. List of enriched pathways based on Reactome pathway analysis of the 602 up-regulated genes from the initial 707 DEG list.
(XLS)

S3 Table Methylation status of genes in the 707 DEG list. Genes that are significantly differentially methylated from the survival based DEG list.
(XLS)

S4 Table Multivariate testing of signature score with tumor grade, patient sex, and age. Cox proportional hazard model used to take into account tumor grade, sex, and age. Total of 237 samples from ICGC pancreatic cancer dataset were used.
(XLS)

S5 Table AJCC stage, differentiation status, and presence of vascular invasion as noted in the pathology reports of human pancreatic tumor samples (n = 10) used calculated the composite H-score. (XLSX)
