# Supplementary figures and images for: Pancreatic cancer survival analysis defines a signature that predicts outcome
Source: PLoS One. 2018 Aug 9;13(8):e0201751. doi: 10.1371/journal.pone.0201751 (PMC6084949; doi:10.1371/journal.pone.0201751)

Survival Time vs. Survival Quantile

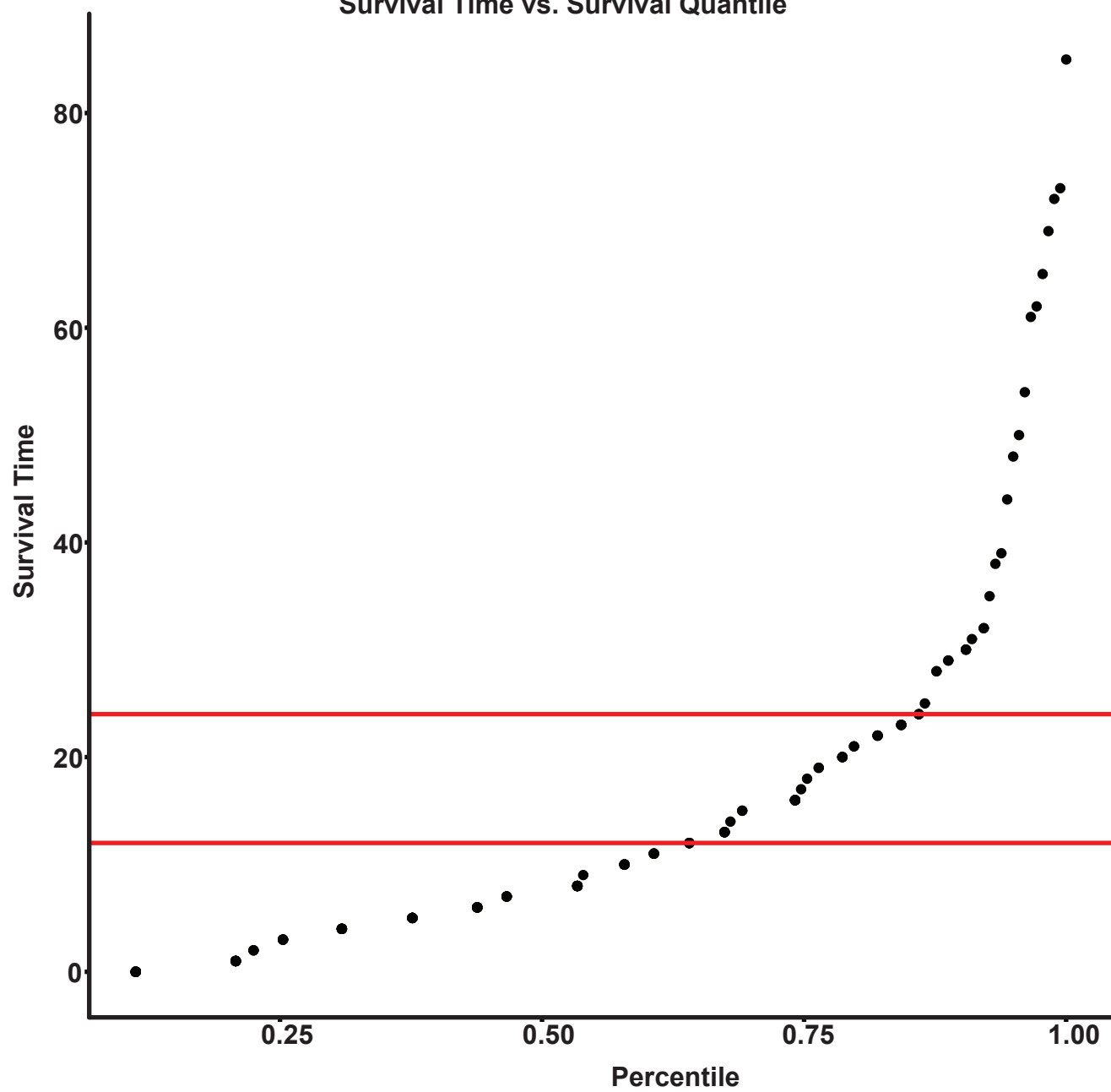

Supplement: S1 Fig — (PDF) [file pone.0201751.s001.pdf]

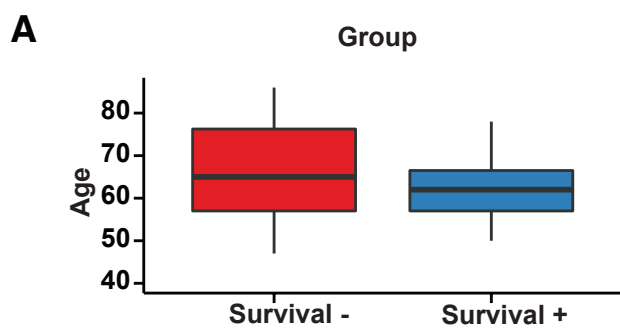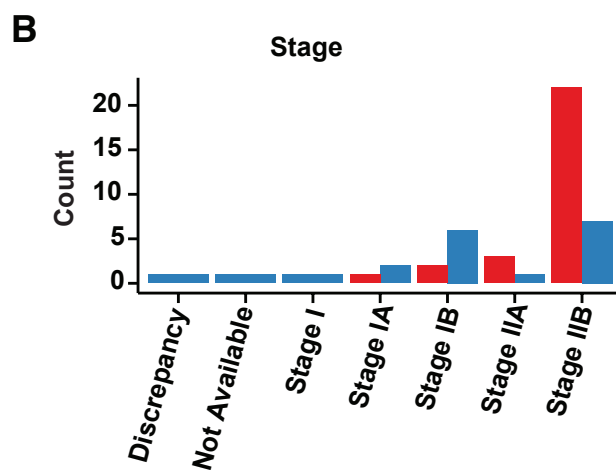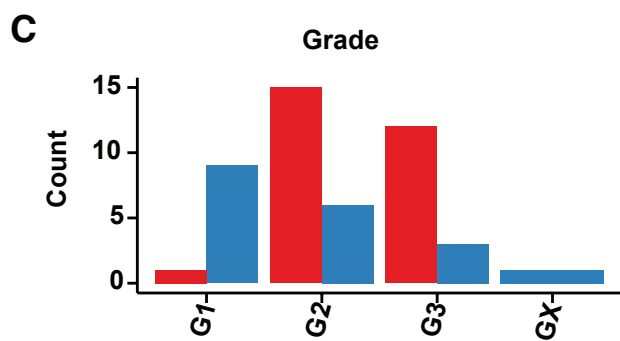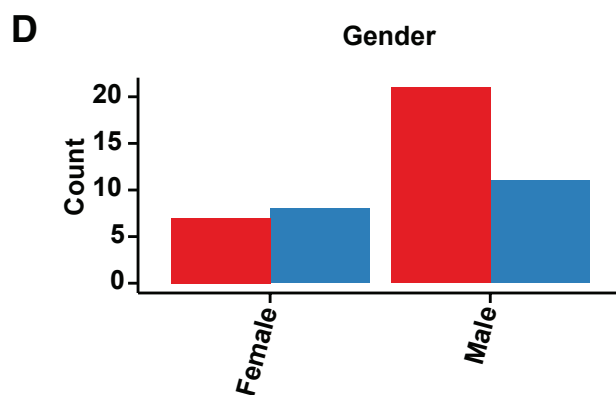

Group ■ Survival - ■ Survival +

Supplement: S2 Fig — Clinical summary information of age, stage, grade, and gender differences between the Survival- and Survival+ groups. Stage and tumor grade were significantly different (p-value = 0.01 and 6x10^-4^, respectively). (PDF) [file pone.0201751.s002.pdf]

A

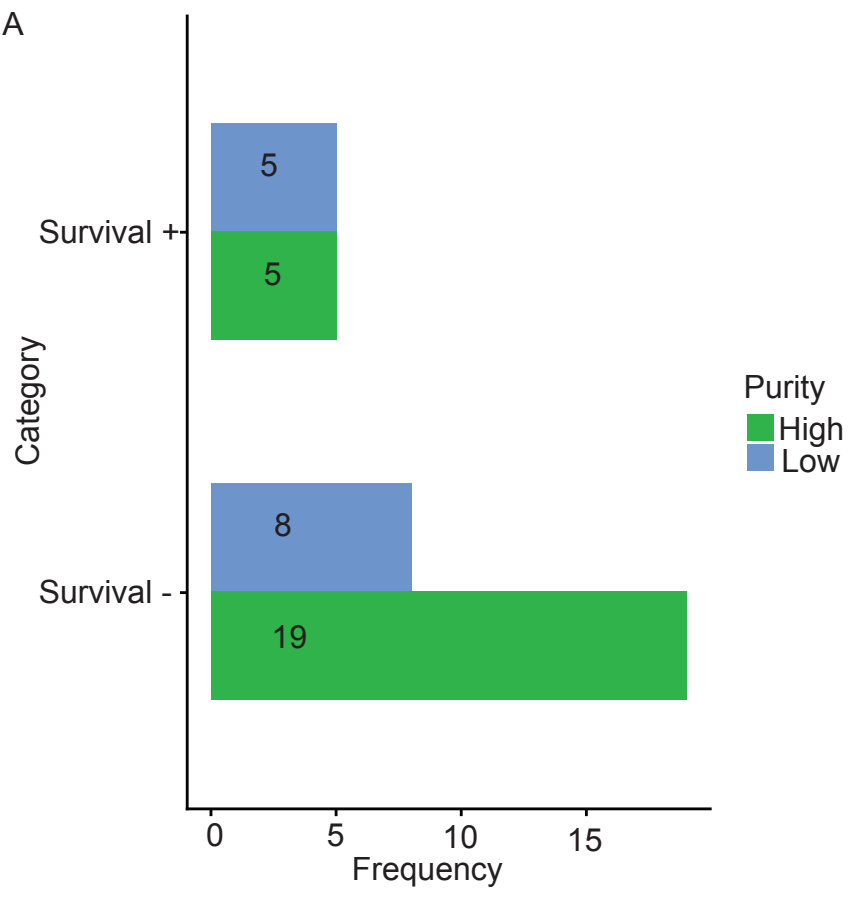

B

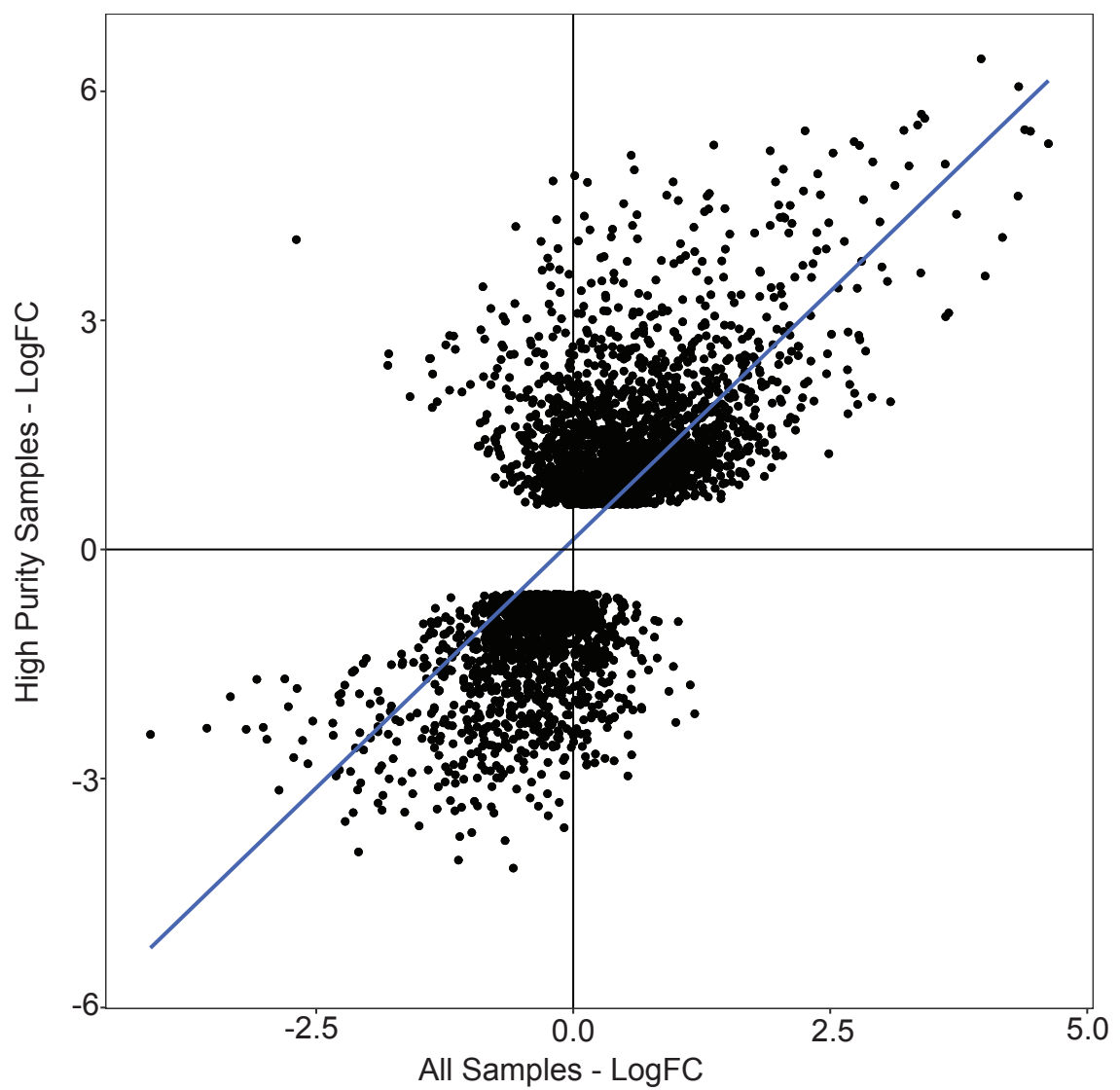

Supplement: S3 Fig — (A) Bar graph showing breakdown of the available high and low tumor cell purity samples present within the Survival + and—groups. (B) Correlation of Survival associated DEGs in high purity PAAD samples to all PAAD samples in the survival cohort. Correlation between DEGs was 0.68 with p < 2.2 x 10^−16^. (PDF) [file pone.0201751.s003.pdf]

**A**

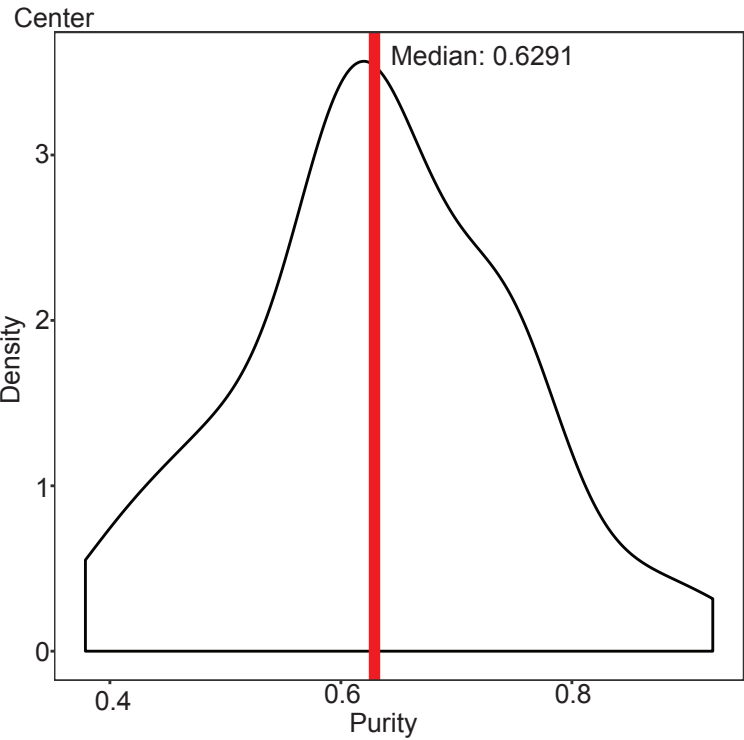

**B**

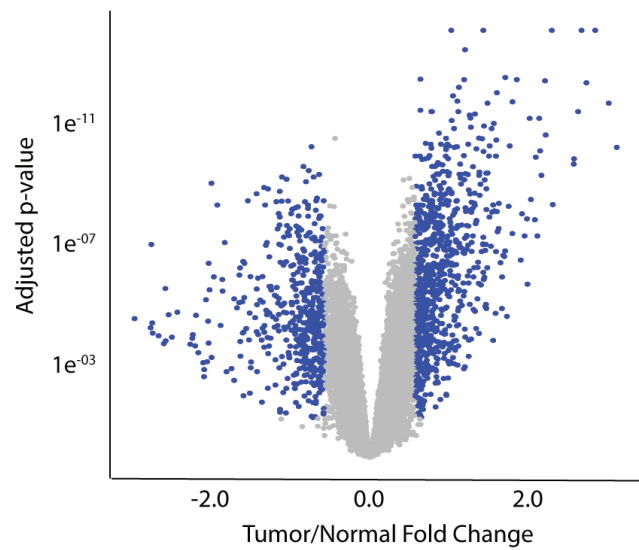

**C**

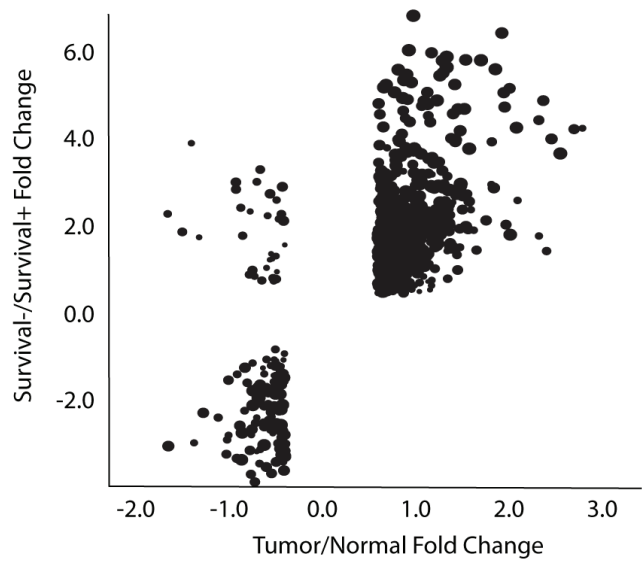

Supplement: S4 Fig — (A) Analysis of tumor cell purity in GSE28735 using the ESTIMATE algorithm. (B) Volcano plot of tumor versus normal pancreatic tissue from the GSE28735 dataset. (C) Scatter plot of log fold change from tumor versus normal comparison and log fold change from survival analysis with signature genes selected. (PDF) [file pone.0201751.s004.pdf]

A

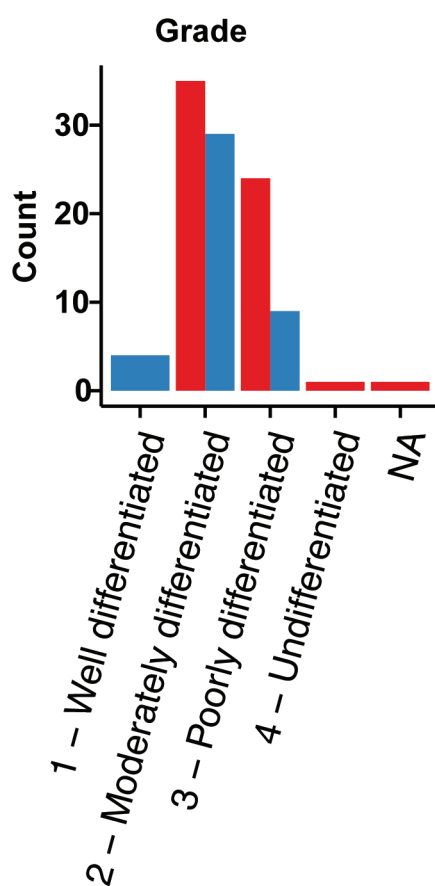

B

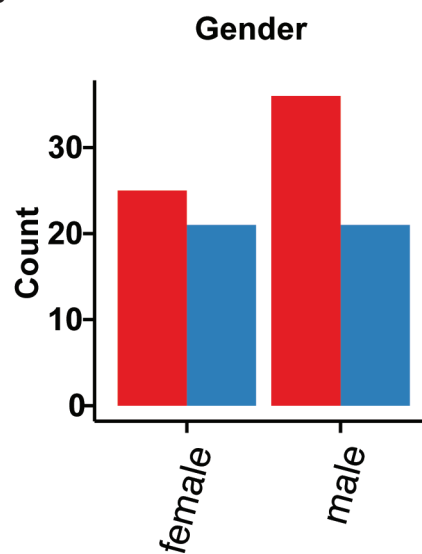

C

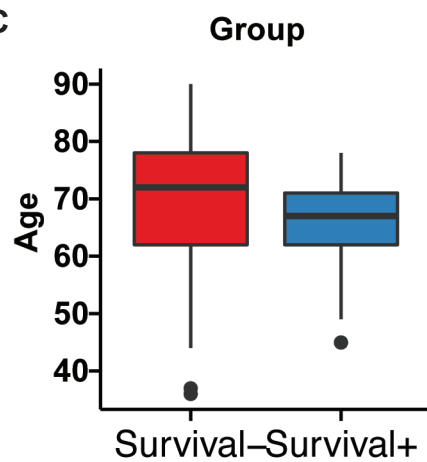

D

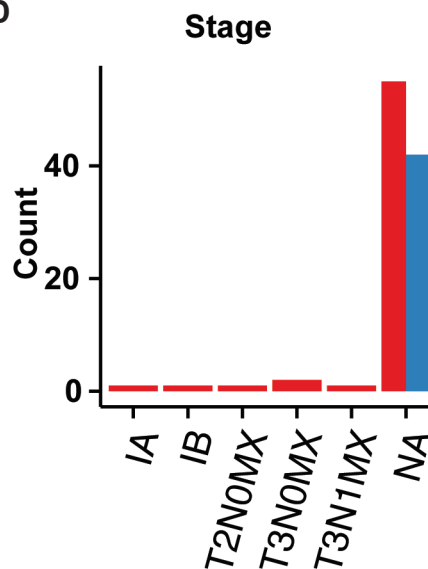

Group: ■ Survival - ■ Survival +

Supplement: S5 Fig — Clinical summary information of (A) grade, (B) gender, (C) age, and (D) stage for the 103 samples in the Australian pancreatic cancer dataset from the ICGC database. (PDF) [file pone.0201751.s005.pdf]

A

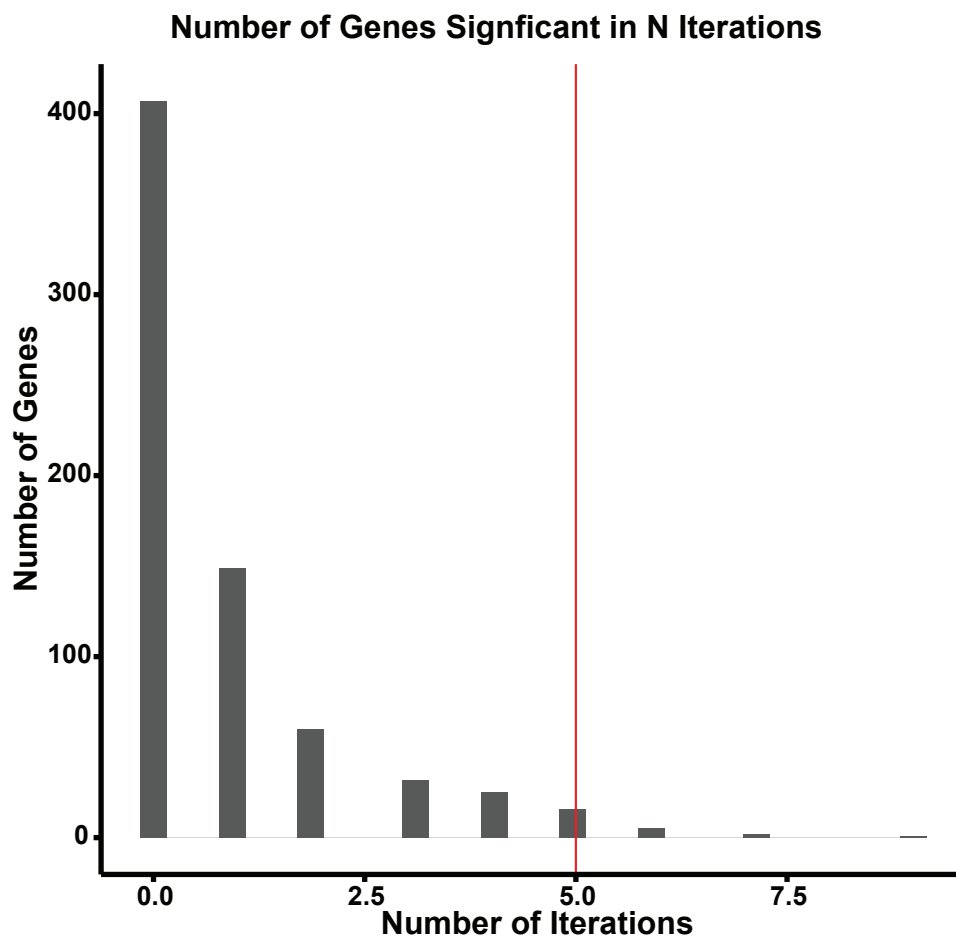

B

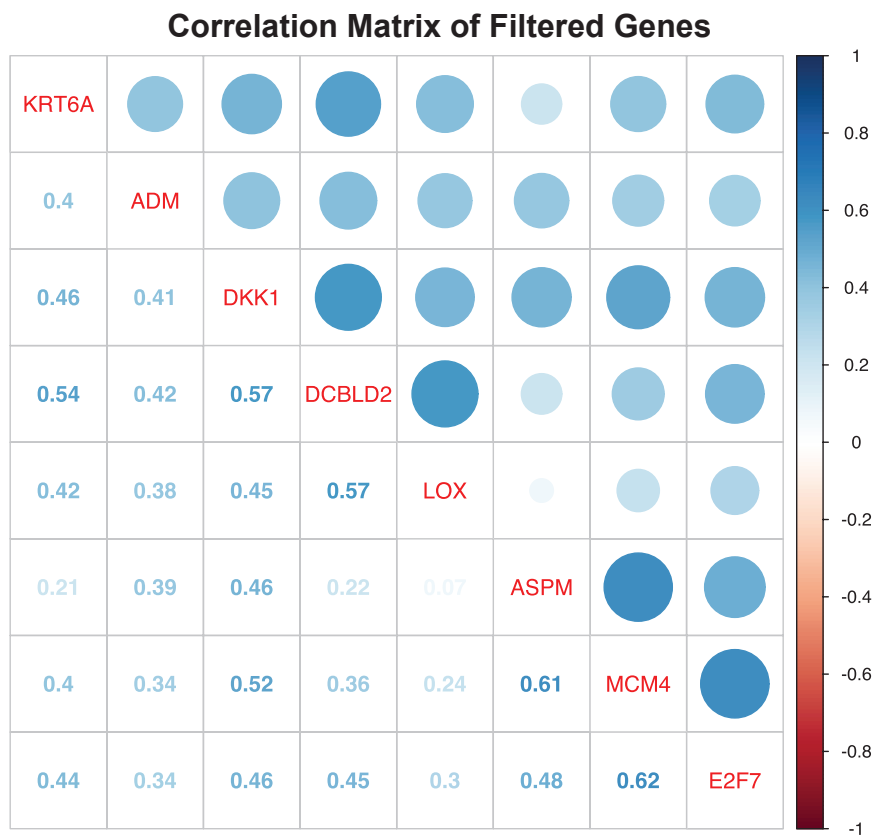

Supplement: S6 Fig — (A) Number of genes significant in N iterations of differential expression analysis. (B) Correlation matrix of genes highly predictive of survival to be considered for signature. (PDF) [file pone.0201751.s006.pdf]

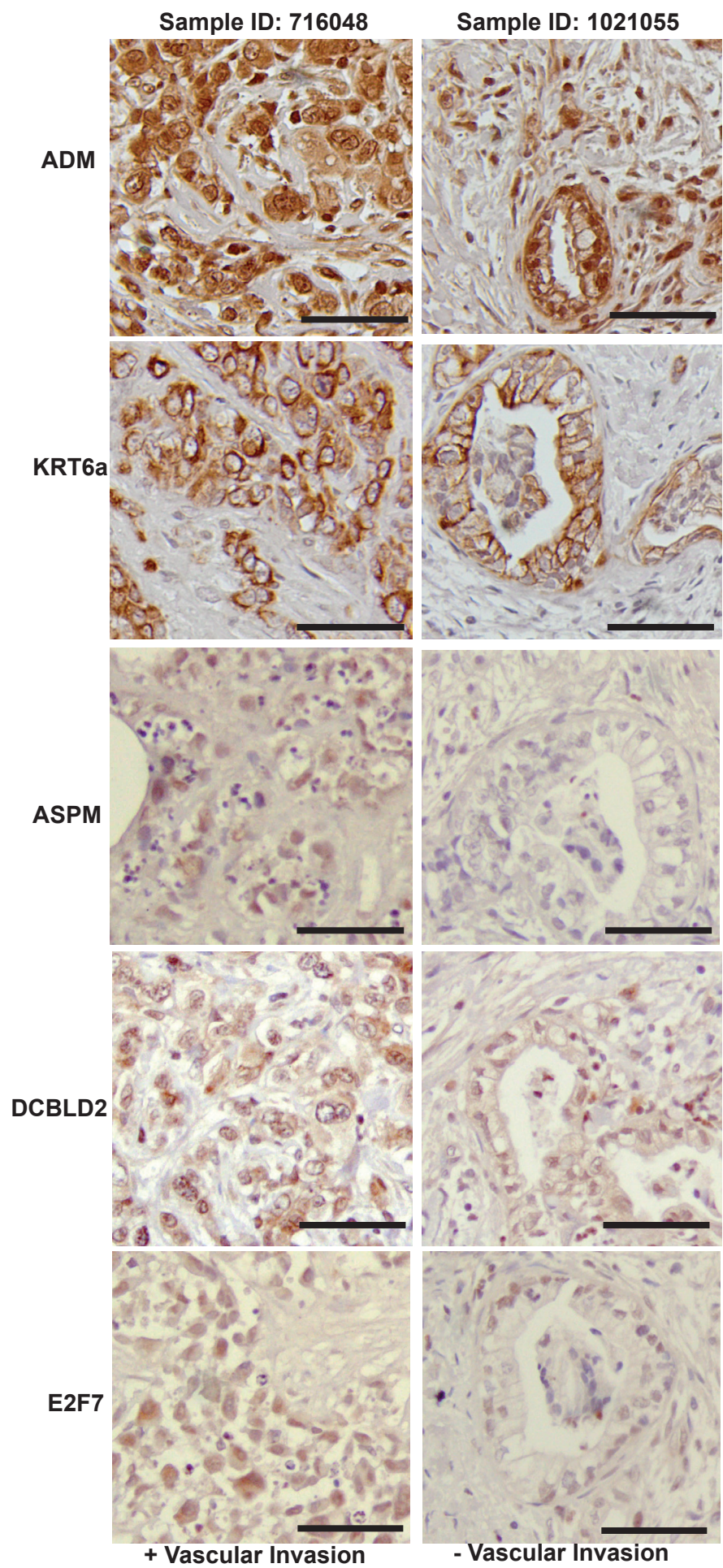

Supplement: S7 Fig — Left column represents tumor tissue from sample ID 716048 which had evidence of vascular invasion. Right column represents tumor tissue from sample ID 1021055 which did not have evidence of vascular invasion. Scale bar is 50μm for all images. (PDF) [file pone.0201751.s007.pdf]
