# Supplementary material for: Pancreatic cancer survival analysis defines a signature that predicts outcome
Source: PLoS One. 2018 Aug 9;13(8):e0201751. doi: 10.1371/journal.pone.0201751 (PMC6084949; doi:10.1371/journal.pone.0201751)
Supplement: S8 Fig — Comparison of null distribution of AUC values to AUC of pancreatic survival signature based on Pancreatic ICGC (left), GSE57495 (middle), GSE71729 (right) datasets. (PDF) [file pone.0201751.s008.pdf]

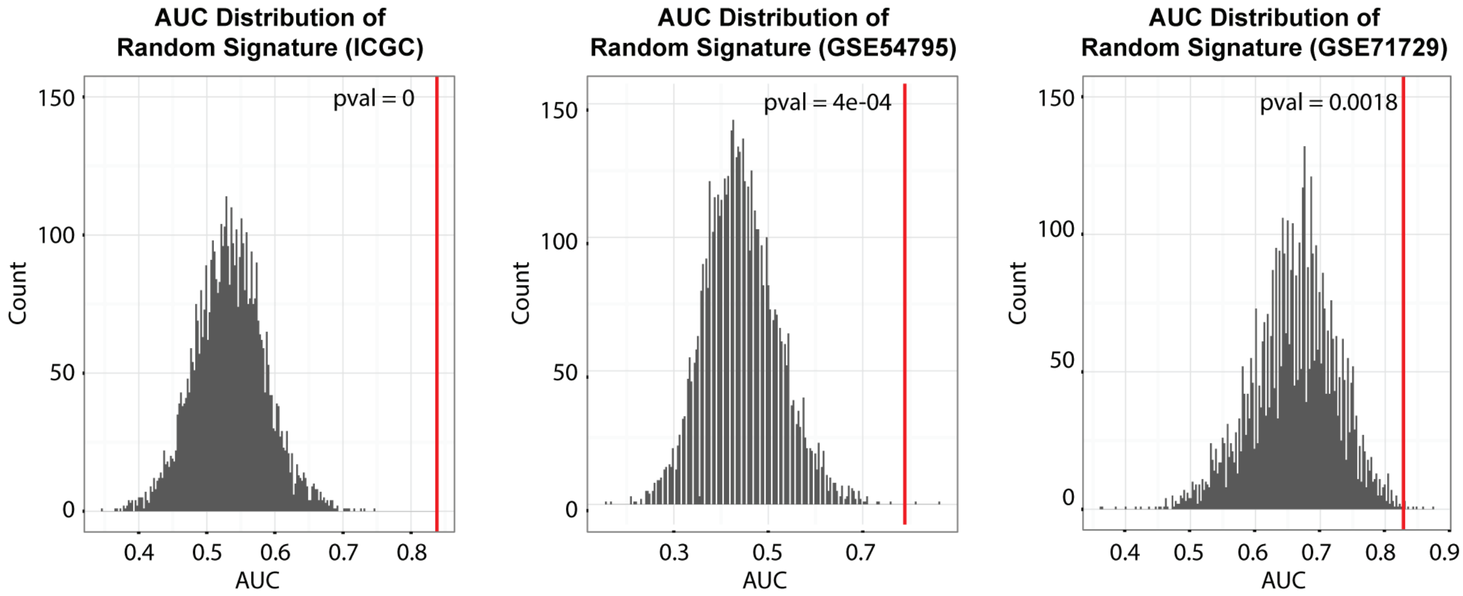

AUC Distribution of
Random Signature (ICGC)
pval = 0
Count
150
100
50
0
0.4
0.5
0.6
0.7
0.8
AUC
AUC Distribution of
Random Signature (GSE54795)
pval = 4e-04
Count
150
100
50
0
0.3
0.5
0.7
AUC
AUC Distribution of
Random Signature (GSE71729)
pval = 0.0018
Count
150
100
50
0
0.4
0.5
0.6
0.7
0.8
0.9
AUC
